# Dendrimer-based drug delivery systems: history, challenges, and latest developments

**DOI:** 10.1186/s13036-022-00298-5

**Published:** 2022-07-25

**Authors:** Juan Wang, Boxuan Li, Li Qiu, Xin Qiao, Hu Yang

**Affiliations:** 1 College of Biomedical Engineering, Sichuan University, Chengdu, 610065 Sichuan China; 2 Tianjin Key Laboratory of Biomedical Materials, Key Laboratory of Biomaterials and Nanotechnology for Cancer Immunotherapy, Institute of Biomedical Engineering, Chinese Academy of Medical Sciences & Peking Union Medical College, Tianjin, 300192 China; 3 Linda and Bipin Doshi Department of Chemical and Biochemical Engineering, Missouri University of Science and Technology, Rolla, MO 65409 USA

**Keywords:** Dendrimer, Drug delivery, Dendrimer hydrogel, Microgel, Nanogel

## Abstract

Since the first dendrimer was reported in 1978 by Fritz Vögtle, dendrimer research has grown exponentially, from synthesis to application in the past four decades. The distinct structure characteristics of dendrimers include nanoscopic size, multi-functionalized surface, high branching, cavernous interior, and so on, making dendrimers themselves ideal drug delivery vehicles. This mini review article provides a brief overview of dendrimer’s history and properties and the latest developments of dendrimers as drug delivery systems. This review focuses on the latest progress in the applications of dendrimers as drug and gene carriers, including 1) active drug release strategies to dissociate drug/gene from dendrimer in response to stimuli; 2) size-adaptive and charge reversal dendrimer delivery systems that can better take advantage of the size and surface properties of dendrimer; 3) bulk and micro/nano dendrimer gel delivery systems. The recent advances in dendrimer formulations may lead to the generation of new drug and gene products and enable the development of novel combination therapies.

## Introduction

### Dendrimer: a brief history and its unique properties

The word “dendrimer” derives from a Greek phrase of “dendron”, which means tree or meros or branch [1]. As early as 1978, Buhleir and coworkers synthesized and reported the first “cascade” and “nonskid-chain-like” molecules with molecular cavity topologies, which later were recognized as the early forms of dendritic polymers [2]. From 1979 to 1985, Donald A. Tomalia and his coworkers at the Dow Laboratories made a breakthrough in the development of dendrimers [3]. They produced polymers with a central, hollow core and tendrils that branched outward, one from another, in a precise, predictable manner, which Tomalia called dendrimers [4]. These two scientific groups contributed to the early history of dendrimers. Up to now, more than 100 dendritic structures have been reported, in which polyamidoamine (PAMAM) dendrimers, polypropyleneimine (PPI) dendrimers, as well as polyamide-, polyether-, polyester-, and phosphorus-based dendrimers are some of the most commonly recognized dendritic families [2, 3, 5–15] (Table 1). Furthermore, thanks to the development of various synthetic strategies, including efficient orthogonal click chemistry and multicomponent reaction (MCR), many new dendrimers with the efficient synthetic process and structure diversity have emerged [16–19]. All the development has promoted the flourishing of dendrimers and their applications in chemistry, materials, and biological and medical science.

**Table 1:** Several commonly recognized dendrimers

Dendrimer Type	Cascade	PAMAM	PPI	Polylysine	Polyester	Phosphorus
First Reported	Vögtle *et al. *in 1978 [2]	Tomalia *et al. *in 1985 [3]	Meijer *et al. *in 1993 [12]	Denkewalter *et al.* in 1981 [13]	Frechet *et al. *in 2002 [14]	Majoral *et al. *in 1994 [15]
Typical Chemical Structure						

**Fig. 1:**
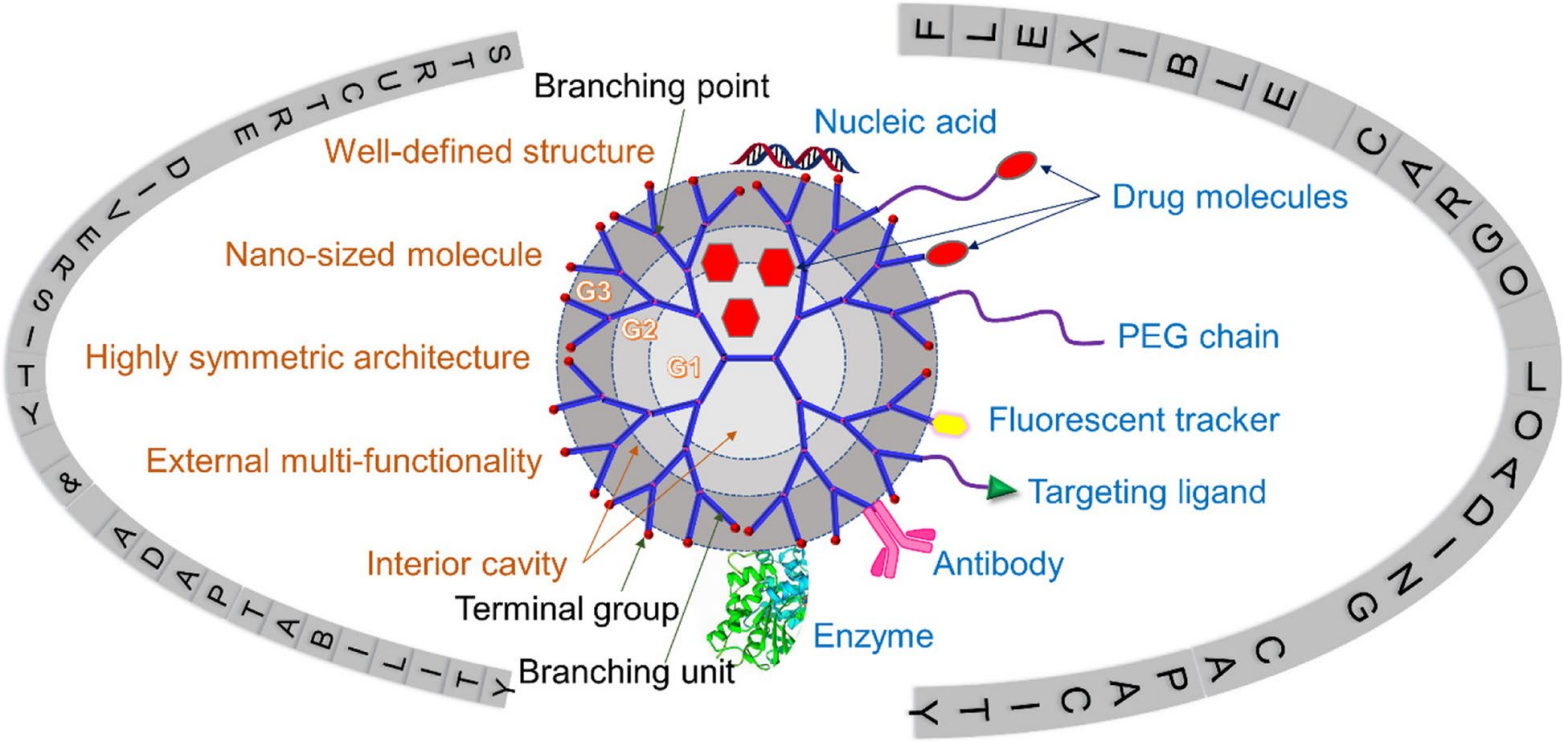
Structure diversity, adaptability and flexible cargo loading capacity of dendrimers. G1, G2, and G3 represent generation 1, 2, and 3, respectively

Dendrimer is different from traditional linear polymers by its mono-dispersity, high symmetricity, and surface polyvalency [20, 21]. The repeated growth reactions during dendrimer synthesis lead to higher generation and degree of branching, eventually forming a three-dimensional spherical structure [20]. The distinct synthetic process makes dendrimer possess a well-defined core–shell architecture and narrow polydispersity [1, 9]. The size, surface charge, peripheral functional groups, and solubility of dendrimer could also be controlled by the synthetic process [1]. For instance, higher-generation dendrimers possess bigger size, larger interior cavity, and more terminal functional groups. In addition to the routine analytical methods, including nuclear magnetic resonance (NMR), gel permeation chromatography (GPC), dynamic light scattering (DLS), high-performance liquid chromatography (HPLC), etc., characterization including electrophoretic and mass spectroscopy measurements have been developed to detect the mono-dispersity features and the effective charge of dendrimers [22, 23]. By using capillary electrophoresis, the purity, electrophoretic mobility, and molecular charge distribution of various surface-charged PAMAM dendrimer nanoparticles can be assessed [22]. The nanoscopic characteristics of individual PAMAM dendrimers from generations 5 to 10 (G5 to G10) were confirmed by transmission electron microscopy (TEM) reported by Jackson and coworkers in 1998 [24]. The mean diameters increase from 4.3 nm for PAMAM G5 to 14.7 nm for PAMAM G10. Besides the nanoscopic feature, the multivalency of dendrimers makes them amenable to further modification or conjugation with chemical species such as fluorophores, targeting ligands, drugs, and genes (Fig. 1).

### Dendrimers as drug delivery vehicles

**Fig. 2:**
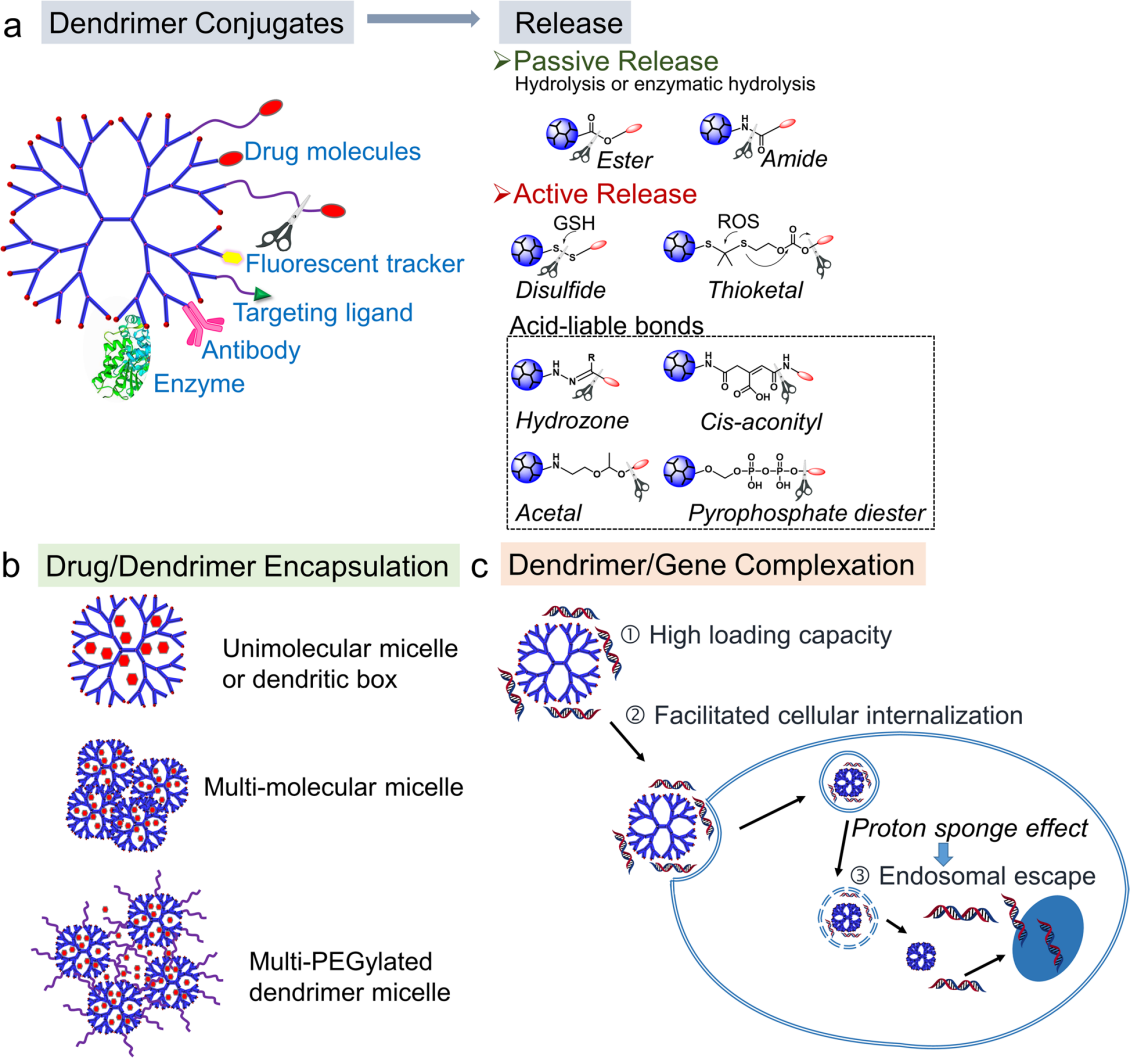
Ways to utilize dendrimers to deliver drugs and genes: **a** dendrimer conjugates and cleavable linkers used to make dendrimer conjugates, **b** three drug/dendrimer encapsulation models, **c** dendrimer/gene complexation, and how cationic dendrimer helps enhance transfection efficiency

Dendrimers have emerged as an important group of nanostructured carriers for the development of nanomedicine to treat various diseases. Because of structural diversity and adaptability, dendrimers have been used to deliver drugs and genes in many different ways (Fig. 1). For instance, dendrimers with a hydrophobic core and a hydrophilic periphery may behave like unimolecular micelles, and they have been utilized to solubilize hydrophobic drugs by entrapping them in the intramolecular cavity [25, 26]. Cationic dendrimers have been extensively applied as non-viral gene carriers [27]. Dendrimer surface groups can be conjugated with drugs and other functional moieties (Fig. 2a) [28, 29]. Conjugating dendrimers with polymers such as polyethylene glycol (PEG), polysaccharide, and polypeptide mainly enhances stability and solubility of the therapeutics to be delivered [30]. PEGylation of dendrimers is a common process through which PEG chains are conjugated to dendrimers, forming a unimolecular micelle [31]. Dendrimer-polysaccharide conjugates are usually adopted to endow the nanomaterials with attractive binding properties and improved compatibility [30, 32, 33]. Polysaccharides, for instance chitosan, hyaluronic acid, cyclodextrin, and dextran have been broadly conjugated to dendrimers [34–37]. A hyaluronic acid conjugated PAMAM dendrimer showed enhanced tumor penetration property due to the strong affinity of hyaluronic acid to CD44 receptors, which are overexpressed on tumor cells and cancer stem cells [35]. He et al. reported a mannose conjugated PAMAM dendrimer for the targeted delivery of liver-x-receptor (LXR) ligands to macrophages as mannose can bind specifically to mannose reporter expressed on macrophage surface [38].

#### Dendrimer-drug conjugates

Dendrimer-drug conjugates could reduce systemic effects and increase efficacy at the targeted site compared with free drugs [25, 39]. It is reported that the half-life of drugs can be increased by conjugating with dendrimers. For instance, the half-life of methotrexate is increased to 24 h from 24 min when conjugated with PAMAM dendrimer [29]. The extended circulating half-life can thus enhance the drug efficacy and lower the frequency of drug administration, improving patient compliance. Solubility of drugs is enhanced when conjugated with dendrimers. For example, paclitaxel’s water solubility is enhanced by 9000-fold when conjugated with PAMAM dendrimer [29]. We previously reported a dendrimer-drug conjugate of DenTimol and studied its therapeutic efficacy for glaucoma treatment [40]. An antiglaucoma drug precursor, (S)-4-[4-(oxiranylmethoxy)-1,2,5-thiadiazol-3-yl]morpholine (OTM), was conjugated to PAMAM dendrimer surface through a PEG spacer. DenTimol is efficient at crossing the cornea due to the good mucoadhesive property of dendrimer, and about 8% of the dendrimer-drug permeated through the cornea in 4 h. DenTimol demonstrated a stronger intraocular pressure (IOP) lowering effect than timolol maleate in normotensive adult Brown Norway male rats. A single dose of DenTimol (10 μL of 0.5% w/v timolol) resulted in an IOP reduction by an average of 7.3 mmHg in less than 0.5 h, which was significantly stronger than timolol PBS eye drops.

According to the United Food and Drug Association (FDA), dendrimer-drug conjugates may be classified into new drugs or combinational devices [41]. If drugs can be cleaved from dendrimer-drug conjugates and maintain their initial structure, this bureaucratic regulation issue could be avoided. Therefore, it is important to examine the release of drugs from dendrimer-drug conjugates (Fig. 2a) [28]. The most facile way is to conjugate drug to dendrimer via a cleavable or stimuli-liable bond. Disulfide and thioketal linkers can be cleaved by glutathione and reactive oxygen species (ROS) in tumor cells, respectively. Therefore, they have been broadly used in designing cleavable dendrimer-drug conjugates [42–45]. pH-responsible linkers, especially acid-liable bonds (e.g., acetal bond), are also used to make dendrimer-drug conjugates for cancer therapy due to the acidic tumor microenvironment [46]. We have constructed a dendrimer-camptothecin-based hydrogel drug delivery system [47]. In this novel drug delivery system, a pH-controlled self-cleaving release of camptothecin (CPT) is realized via the ammonolysis of ester bonds between the dendrimer and CPT. The controlled self-cleaving release mechanism significantly prolonged CPT release and thus enhanced tumor inhibition.

#### Drug/dendrimer encapsulation

It is broadly recognized that the interior hydrophobic cavities of dendrimers can accommodate hydrophobic drugs (Fig. 2b) [25]. The encapsulation of hydrophobic drugs in dendrimer cavities leads to an increase in water solubility of the hydrophobic drugs [48]. This guest–host encapsulation of drug/dendrimer makes dendrimer behave as “unimolecular micelle” or “dendritic box” [25]. Low-generation dendrimers have limited drug loading capacity due to a small inner space. PEGylated dendrimers possess enhanced drug loading capacity because of the agglomeration of PEGylated dendrimer molecules [31]. PEGylated dendrimers could also promote enhanced permeation and retention effect (EPR) of the encapsulated drugs [25].

#### Dendrimer/gene complexation

Amine-terminated PAMAM dendrimers have been broadly used as gene transfection vectors (Fig. 2c) [49–51]. Compared with branched polyethylenimine (PEI), PAMAM dendrimers show higher biocompatibility and larger nucleic acid loading capacity [52]. The nanoscopic size, spheroidal shape, and cationic surface of PAMAM dendrimers facilitate cellular uptake of the complexed nucleic acids [53]. In addition, the proton sponge effect of PAMAM dendrimers helps endosomal escape, which is a critical step for augmenting transfection efficiency [54]. We synthesized G4-FA and tested it as a vector for local delivery of siRNA against vascular endothelial growth factor A (siVEGFA) in a xenograft tumor mouse model of head and neck squamous cell carcinomas [55]. G4-FA facilitated the siVEGFA delivery, promoted its tumor-specific uptake, and substantially inhibited tumor growth of head and neck cancer. Compared with siVEGFA group, two doses of G4-FA/siVEGFA intratumorally administered eight days apart resulted in a significant inhibition on tumor growth, accompanied with profound reduction in angiogenesis.

Dendrimers are often decorated with additional functional moieties such as peptides to overcome intracellular gene delivery barriers [56]. Our group recently reported PAMAM dendrimer complexed with a synthetic diblock nuclear-localization sequence peptide (NLS) and used it for gene delivery [57]. The complexed NLS promoted the nuclear translocation of the entire dendrimer/nucleic acid polyplex and then destabilized the association between PAMAM and plasmid in the nucleus, eventually leading to enhanced gene transfection. Similar to gene transfection, cationic dendrimers are also able to bind with other negatively charged molecules, such as heparin or polyanions [58]. For instance, the complex of polylysine dendrimer with heparin has the potential to be used as stable anti-angiogenic therapeutics as it neutralizes the anticoagulant activity of heparin in plasma [58].

## Challenges and solutions to dendrimer-based drug and gene delivery

### Challenges

Despite the benefits of dendrimers as drug delivery carriers, some challenges remain to be solved. The size and surface chemistry of dendrimers are closely related to their toxicity and biodistribution [59]. Size limitation is a primary concern. PAMAM dendrimers of generation 5 or lower can be sufficiently eliminated via glomerular filtration in the renal excretion pathway, while the clearance of PAMAM generation 6 and higher rely more on the hepatic clearance pathway [51, 60]. Dendrimers with sizes ranging from 4–10 nm have the ability to interact with nanometric cellular components and have the capacity to overcome the cellular endocytosis barrier [59, 61]. However, PAMAM dendrimers of generation 6 and higher have high costs and severe toxicity [51], therefore the higher generation of PAMAM dendrimers are rarely used. Cationic dendrimers possess high binding capacity with nuclei or anion compounds and facilitate cell internalization [62, 63]. However, cationic dendrimers often encounter nonspecific adsorptions of plasma proteins and accelerated elimination by the reticuloendothelial system [59]. In addition, the intracellular dissociation of dendrimers with nuclei acids is limited [64]. Since the interaction of cationic dendrimers with negatively charged cell membranes can result in the destabilization of biological membrane and thus cause cell lysis, cationic dendrimers generally exhibit higher toxicity, especially at high doses, than neutral or anionic dendrimers [65–67]. Pryor et al. studied the toxicity of PAMAM on embryonic zebrafish models and found that cationic PAMAM generation 6 was statistically more toxic than both neutral PAMAM generation 6 and anionic PAMAM generation 6 at the same concentration [68]. Recent developments that have been made to address the challenges mentioned above are summarized below.

### Size adaptive dendrimer clustered nanoparticles

Strategies have been developed to use dendrimers as building blocks to form larger nanoparticles. For instance, size-switchable or adaptive nanoparticles obtained from dendrimer clusters have attractive features [69, 70]. They are mostly hundreds of nanometers in size and remain stable during blood circulation. After reaching the target tissues, nanoparticles would disintegrate and release individual dendrimers to exert their extraordinary tissue penetration and cell internalization property. Gao et al. constructed size- and charge-adaptive clustered nanoparticles based on the electrostatic interaction between PAMAM and 2,3-dimethyl maleic anhydride modified poly(ethylene glycol)-block-polylysine (PEG-b-PLys) [69]. The clustered nanoparticle consists of PEG chains as the outer layer and complexes of PLy chains with PAMAM as the inner core. The clustered nanoparticles were shown to have longer blood circulation when they were slightly larger than 100 nm (112 nm) and had a slightly negatively charged surface (-2.2 mV) with peripheral PEG chains. Upon arriving at the infected lung tissue, carboxyl groups in the PLy segments switched to amine groups in the acidic microenvironment, disassembling dendrimer and PEG-b-PLys. By taking advantage of small size (6.5 nm) and positive charge (23.8 mV), the released PAMAM dendrimers achieved effective penetration and long-term retention inside biofilms.

Wang et al. fabricated size-switchable nanoparticles through self-assembly of linear-dendritic triblock copolymer poly(ethylene glycol)-*b*-poly(ε-caprolac-tone)-polyamidoamine (PEG-*b*-PCL-PAMAM) [70]. A singlet oxygen responsive thioketal linker connected PEG-*b*-PCL and PAMAM segments. The formed nanoparticles had a hydrodynamic size of 118 nm and were shown to accumulate in the tumor through the EPR effect. In the blood vessel extravasation site, irradiation of 660 nm was applied to generate singlet oxygens by the pre-loaded photosensitizer chlorin e6 (Ce6). The produced singlet oxygen not only killed cancer cells but also triggered the cleavage of the thioketal linker, releasing indocyanine green conjugated PAMAM (PAMAM-ICG). Because of its small size, the released PAMAM-ICG penetrated deeper into the tumor for improved photothermal and photodynamic therapy.

### Surface stealth modification during body circulation: charge-reversal dendrimers

PEG is an inert, non-immunogenic, and non-antigenic polymer with excellent water solubility and biocompatibility, and PEG has been approved by FDA [71, 72]. PEGylation of dendrimers is commonly used to shield the cationic surface of dendrimers, reduce their toxicity, and prolong their circulation time [72, 73]. PEGylated PAMAM dendrimers increase the systemic circulation time by reducing the unspecific absorption and thus increase the accumulation in the target tissues [74]. Nevertheless, such a strategy impedes dendrimer tissue penetration and subsequent cell internalization. PEGylated dendrimers can also enhance the solubilization of hydrophobic drugs, extend the flexibility for dendrimer conjugation, improve DNA transfection, and promote tumor targeting [72, 75]. However, long-term administration of PEGylated formulations leads to the accumulation of PEGs within tissues, leading to potential tissue toxicity and adverse effects [76–78]. In addition, increasing evidence shows that the excessive use of PEG may lead to the production of PEG antibodies in the body, causing accelerated blood clearance (ABC) and shortening blood circulation time of PEGylated materials [79–81].

Further research is thus warranted to bring these promising nanomaterials from the bench to the bedside [72]. Acetylation of amine groups can decrease the positive surface charges of PAMAM dendrimer and thus reduce its toxicity. Waite et al. reported that modest acetylation (approximately 20% degree of amine was acetylated) of PAMAM could maintain the siRNA delivery efficiency while reducing the toxicity [82]. Recently, zwitterionic modified nanomaterials have been reported to have a better antifouling property than PEGylated counterparts [83]. Wang et al. reported a zwitterionic nanocarrier self-assembled from a Janus dendrimer [84]. The Janus dendrimer comprises two distinct dendrons: one is hydrophilic and zwitterionic end-group functionalized, the other is modified with positively charged arginine groups and hydrophobic moieties. This Janus dendrimer could self-assemble into a larger nanoparticle with zwitterionic moieties as the outer layer. It was shown to repel proteins and gain prolonged circulation in the blood compared to the PEGylated nanocarriers.

Another idea is to deactivate the cationic surface charges of dendrimers during blood circulation to minimize the nonspecific cellular uptake and adsorption and then reactivate the positive surface charges once inside the target tissues or cells. This is the basis for making charge-reversal or charge-switchable dendrimers. Cues including temperature, pH, osmotic pressure, and biological signals may be utilized to reverse charges on the dendrimer surface [85–91]. We summarize the recently reported charge-reversal chemistries in Table 2. In general, the transformation from negative charge to positive charge is conducive to the stability of the materials in the blood circulation and their endocytosis, while the transformation from positive charge to negative/neutral charge is helpful to improve the release of nucleic acids (which will be discussed in the following section).

**Table 2:** Surface charge-reversal chemistries

Charge Reversal	Chemistry	Stimuli	Refs
Negtive ⬇ Positive		pH	[45]


		Enzyme: γ-glutamyl transpeptidase (GGT)	[42, 85, 86]
Positive ⬇ Negtive		Enzyme: Glutathione (GSH)	[91]
		ROS	[90]
Positive ⬇ Neutral		pH	[92]

### Intracellular controlled gene release

Prompt separation of nucleic acids from their carrier following cellular internalization and endosomal escape is essential to achieving high gene transfection. It is desirable to design a spatiotemporally controllable gene delivery vehicle to release nucleic acids intracellularly. The charge-reversal dendrimers are ideal candidates (Table 2). Wang et al. reported a gene delivery system by using the charge-reversal strategy [91]. Deoxyribonucleic acid (DNA) was electrostatically condensed by a positively charged poly{N-[2-(acryloyloxy)ethyl]-N-[p-(2,4-dinitrophenoxy)benzyl]-N,N-diethyl ammonium chloride} (PADDAC) polymer and coated with a liposome layer to maintain the stability in blood circulation. After cell internalization, the liposomal layer degraded in lysosomes, resulting in the exposure of the PADDAC/DNA complex. The over-expressed glutathione in tumor cells triggered the charge-reversal of PADDAC moieties from positive to negative for DNA release. We recently reported a heterogeneous dendrimer derivative (G3-acetal-NH_2_) having an acid-responsive charge reversal periphery [92]. The positive surface charged G3-acetal-NH_2_ has a similar pMAXGFP plasmid condensing capacity to that of native PAMAM G4. After cell uptake, the cleavage of the acetal groups was triggered in the weakly acidic endosome environment, and the surface of the dendrimer turned from amine-terminated to hydroxyl-terminated. The reduction in the dendrimer surface charge resulted in the release of the genetic payload and enhanced GFP protein expression.

## Our solution: hierarchically structuring dendrimers into dendrimer gel

### Dendrimer hydrogel

**Fig. 3:**
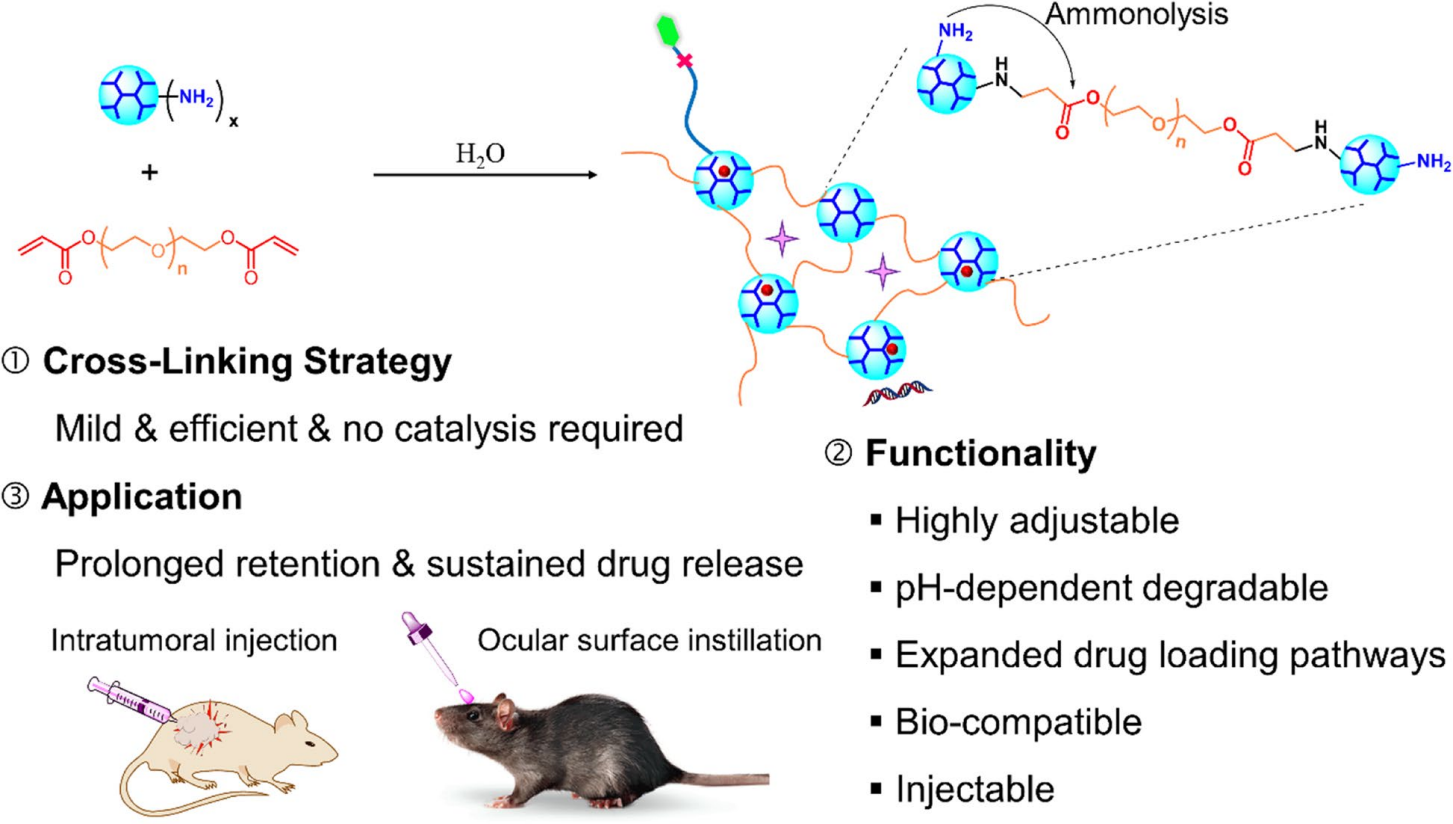
A homogeneous dendrimer hydrogel: cross-linking strategy, tunable functionality, and application for local drug delivery system

When local administration is adopted, such as ocular topical instillation or intratumoral injection, issues associated with the systemic circulation are no longer a concern. But it is of paramount importance to retain the formulation locally. Hydrogels, especially injectable hydrogels, benefiting from their three-dimensional network structure and adhesion property, have been broadly served as drug depots for prolonged drug release [93, 94]. The multifunctionality and well-defined nanostructure make dendrimer an ideal building block for producing three-dimensional cross-linked networks, named dendrimer hydrogel (DH) [95–97]. Many chemical and physical ways were developed to cross-link dendrimers to form hydrogels [97–103]. Recently, we developed a DH platform using the aza-Michael addition cross-linking strategy and explored its application as local drug delivery system (Fig. 3) [97]. Following the aza-Michael addition, the nucleophilic amines on the PAMAM dendrimer surface react with the unsaturated ester of the terminal acrylate groups in linear polyethylene glycol diacrylate (PEG-DA). This dendritic-linear aza-Michael addition cross-linking strategy is a green approach as it proceeds efficiently in aqueous media at room temperature without the use of a catalyst. Since the synthesis of PAMAM dendrimer is also based on the aza-Michael addition between amine and acrylate groups, the formed gel has similar chemical linkages to the original dendrimers. The degree of cross-linking and gel properties can be adjusted readily by controlling reactant concentration or amine group density on the dendrimer surface.

Forming DHs expands the structural diversity of dendrimers and greatly enriches the ways of using dendrimers to deliver drugs and genes. In addition to the delivery methods discussed above, drugs and/or genes can be in-situ embedded in the hydrogel network. Un-reacted amines on the dendrimer surface can accelerate the ammonolysis of the ester bonds to make the hydrogel degradable. What is more interesting is that the ammonolysis process is pH-dependent: a more acidic environment tends to slow down the ammonolysis and extend gel degradation [104]. Importantly, cross-linking dendrimer with PEG makes the cross-linked structure more biocompatible compared to free dendrimers in equivalent molar qualities [97, 105].

We tested this DH as a platform for intratumoral drug delivery to treat head and neck cancer. We embedded the anticancer drug fluorouracil in the DH and tested its in vitro release and in vivo tumor inhibition activities [97]. Injectable fluorouracil loaded DH formulation efficiently inhibited tumor growth following intratumoral injection. Compared to the terminal tumor volume of the PBS group, fluorouracil-loaded DH formulation reduced tumor volume by four-fold following 3-week treatment. We also designed a DH formulation to deliver the anticancer drug CPT via conjugation [47]. In this new formulation, CPT was covalently grafted to PAMAM dendrimer. The dendrimer-CPT conjugates were then cross-linked with PEG-DA to form DH (DH-G3-CPT). Similarly, ammonolysis of ester bonds underlies both CPT release and hydrogel degradation. This novel DH drug delivery system realized a significantly prolonged drug release over a period of 6 days in the acidic tumor site, while a complete release of CPT from this DH at neutral buffer only took 4 days. The sustained release thus led to an excellent tumor inhibition effect following intratumoral injection in a mouse model of head and neck cancer.

Glaucoma has become the leading cause of irreversible blindness worldwide [106]. Eye drop instillation is the most broadly used administration route to deliver drugs to treat glaucoma [107]. DHs have been tested for topical delivery of antiglaucoma drugs [41, 102, 108]. We developed a mildly cross-linked DH as eye drops by choosing the antiglaucoma drug brimonidine tartrate as a model drug [109]. This antiglaucoma drug formulation has both flowability and adhesion due to the moderate cross-linking density. A significantly sustained brimonidine release is also realized. This loosely three-dimensional network of the DH formulation enables a two-phase drug release kinetics: a burst release within the first 6 h allows the drug to rapidly reach an effective concentration, and a subsequent sustained release continuously supplies the drug to maintain the therapeutic drug level for 48 h. The corneal permeability of brimonidine by using this mildly cross-linked DH increased by two-fold. This sustained release and enhanced corneal permeation make the DH formulation have great potential to improve the efficacy of antiglaucoma drugs under topical instillation.

### From dendrimer hydrogel to micro/nano gel: a more precise way to hierarchically use dendrimers as building blocks

**Fig. 4:**
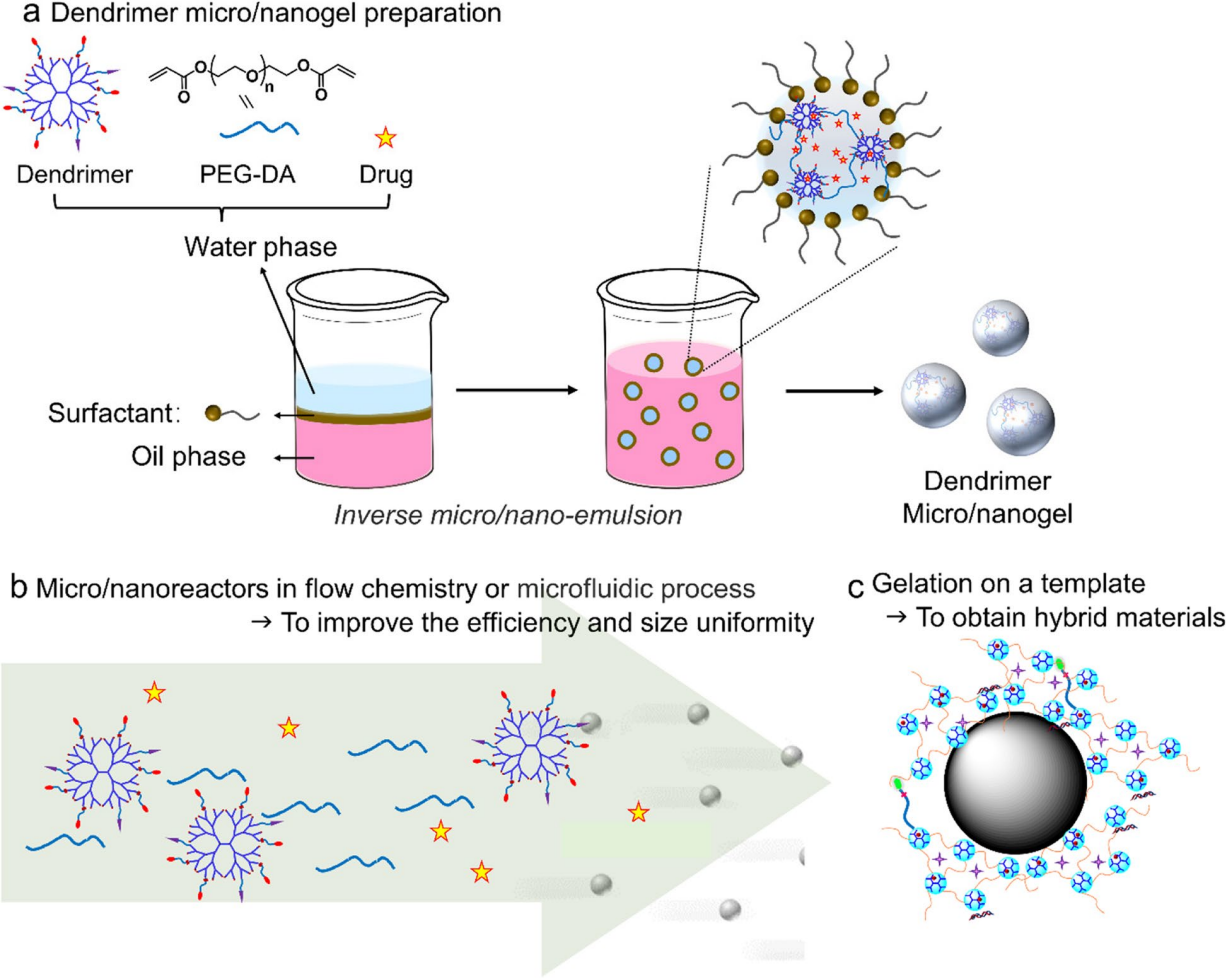
The combined aza-Michael addition cross-linking strategy with inverse micro/nano-emulsion technology to produce dendrimer micro/nanogel (**a**) and future possible preparations to obtain dendrimer micro/nanogel by utilization of microfluidic technology or flow chemistry (**b**) and template methods (**c**)

DHs have shown promising structural features for local drug delivery. We further exploited the preparation process of DH and studied whether DH on a micro/nanoscale could be made. We combined the aza-Michael addition cross-linking strategy with the inverse micro/nano-emulsion method (Fig. 4). All the reactants are dissolved in water first and then converted into micro/nanodroplets in a continuous organic phase in the presence of surfactants. The gelation reaction between dendrimer and PEG-DA occurs in the micro/nanodroplets. We have successfully prepared micro and nanogel particles with size range from several micrometers to hundreds of nanometers by changing preparation parameters [110]. The dendrimer microgel we have prepared (3–5 μm) shows high loading of the hydrophobic drug CPT, zero-order sustained release kinetics, and good cell internalization with excellent cytocompatibility [110]. We modified parameters to make dendrimer nanogels and tested them for topical delivery of antiglaucoma drugs [111]. We found that dendrimer nanogels maximized the utility of the structural features of existing dendrimer and hydrogel ocular drug delivery systems in terms of cytocompatibility, degradability, drug release kinetics, corneal permeability, and sustained IOP-lowering efficacy. The IOP reduction treated by brimonidine tartrate-loaded nanogels was four-fold deeper than that treated by free brimonidine tartrate following 7 days of daily dosing.

Dendrimer micro/nanogels integrate the features of dendrimer, hydrogel, and micro/nanoparticles [111]. They provide a new platform with complex functionality for drug and gene delivery. Different formulation preparation techniques can be combined with dendrimer gelation chemistries. For instance, microfluidic technology or flow chemistry may help produce dendrimer microgels with uniform size as well as increase production efficiency. The adoption of the template preparation method may be used to prepare dendrimer micro-nano gels with better control over structure and enable the production of hybrid materials.

## Conclusions

In this mini review article, we briefly reviewed the history of dendrimers and their features for drug and gene delivery. Dendrimers have been widely adopted as drug/gene delivery vehicles in various forms, including dendrimer-drug conjugates, drug/dendrimer encapsulation, dendrimer/gene complexation, and so on. We also reviewed the challenges and solutions to dendrimer-based drug and gene delivery. In particular, we discussed new dendritic structures reported in recent years, including size-switchable and charge-reversal dendrimers, bulk and micro/nano dendrimer hydrogels. In summary, the recent advances in dendrimer formulations may lead to the generation of new drug and gene products and enable the development of novel combination therapies.

## Data Availability

None.

## Abbreviations

ABC: Accelerated blood clearance
Ce6: Chlorin e6
CPT: Camptothecin
DH: Dendrimer hydrogel
DLS: Dynamic light scattering
DNA: Deoxyribonucleic acid
EPR: Enhanced permeation and retention effect
FA: Folic acid
FDA: Food and Drug Association
GPC: Gel permeation chromatography
HPLC: High-performance liquid chromatography
ICG: Indocyanine green
IOP: Intraocular pressure
LXR: Liver-x-receptor
MCR: Multicomponent reaction
NLS: Nuclear-localization sequence peptide
NMR: Nuclear magnetic resonance
OTM: (S)-4-[4-(oxiranylmethoxy)-1,2,5-thiadiazol-3-yl]morpholine
PADDAC: Poly{N-[2-(acryloyloxy)ethyl]-N-[p-(2,4-dinitrophenoxy)benzyl]-N,N-diethyl ammonium chloride}
PAMAM: Polyamidoamine
PEG: Polyethylene glycol
PEG-*b*-PCL-PAMAM: Poly(ethylene glycol)-*b*-poly(ε-caprolactone)-polyamidoamine
PEI: Polyethylenimine
PLy: Polylysine
PPI: Polypropyleneimine
ROS: Reactive oxygen species
siVEGFA: SiRNA against vascular endothelial growth factor A
TEM: Transmission electron microscopy

## References

[1] Vögtle F, Richardt G, Werner N, Vögtle F, Richardt G, Werner N (2009). Introduction. Dendrimer chemistry: concepts, syntheses, properties, applications.

[2] Buhleier E, Wehner W, Vögtle F (1978). "Cascade"- and "nonskid-chain-like" syntheses of molecular cavity topologies. Synthesis.

[3] Tomalia DA, Baker H, Dewald J, Hall M, Kallos G, Martin S (1985). A new class of polymers: starburst-dendritic macromolecules. Polym J.

[4] Tomalia DA, Fréchet JMJ (2002). Discovery of dendrimers and dendritic polymers: a brief historical perspective. J Polym Sci A Polym Chem.

[5] Liko F, Hindré F, Fernandez-Megia E (2016). Dendrimers as innovative radiopharmaceuticals in cancer radionanotherapy. Biomacromol.

[6] Mintzer MA, Grinstaff MW (2011). Biomedical applications of dendrimers: a tutorial. Chem Soc Rev.

[7] Rosen BM, Wilson CJ, Wilson DA, Peterca M, Imam MR, Percec V (2009). Dendron-mediated self-assembly, disassembly, and self-organization of complex systems. Chem Rev.

[8] Wang D, Astruc D (2013). Dendritic catalysis—basic concepts and recent trends. Coord Chem Rev.

[9] Esfand R, Tomalia DA (2001). Poly(amidoamine) (PAMAM) dendrimers: from biomimicry to drug delivery and biomedical applications. Drug Discov Today.

[10] Idris AO, Mamba B, Feleni U (2020). Poly (propylene imine) dendrimer: a potential nanomaterial for electrochemical application. Mater Chem Phys.

[11] Caminade A-M, Majoral J-P (2004). Nanomaterials based on phosphorus dendrimers. Acc Chem Res.

[12] de Brabander van den Berg EMM, Meijer EW (1993). Poly(propylene imine) dendrimers: large-scale synthesis by hetereogeneously catalyzed hydrogenations. Angew Chem Int Ed Engl..

[13] Robert G. Denkewalter, Jaroslav Kolc, Lukasavage WJ. Macromolecular highly branched homogenerous compound based on lysine units. United States Patent, 4,289,872, Sep.15, 1981.

[14] Gillies ER, Fréchet JMJ (2002). Designing macromolecules for therapeutic applications: polyester dendrimer poly(ethylene oxide) “Bow-Tie” hybrids with tunable molecular weight and architecture. J Am Chem Soc.

[15] Launay N, Caminade A-M, Lahana R, Majoral J-P (1994). A general synthetic strategy for neutral phosphorus-containing dendrimers. Angew Chem Int Ed Engl.

[16] Arseneault M, Wafer C, Morin JF (2015). Recent advances in click chemistry applied to dendrimer synthesis. Molecules.

[17] Deng X-X, Du F-S, Li Z-C (2014). Combination of orthogonal ABB and ABC multicomponent reactions toward efficient divergent synthesis of dendrimers with structural diversity. ACS Macro Lett.

[18] Fan X, Hu Z, Wang G (2015). Facile synthesis of polyester dendrimer via combining thio-bromo “Click” chemistry and ATNRC. J Polym Sci A Polym Chem.

[19] Jee J-A, Spagnuolo LA, Rudick JG (2012). Convergent synthesis of dendrimers via the passerini three-component reaction. Org Lett.

[20] Tomalia DA, Hedstrand DM, Ferritto MS (1991). Comb-burst dendrimer topology: new macromolecular architecture derived from dendritic grafting. Macromolecules.

[21] Abbasi E, Aval SF, Akbarzadeh A, Milani M, Nasrabadi HT, Joo SW (2014). Dendrimers: synthesis, applications, and properties. Nanoscale Res Lett.

[22] Shi X, Majoros IJ, Baker JR (2005). Capillary electrophoresis of poly(amidoamine) dendrimers: from simple derivatives to complex multifunctional medical nanodevices. Mol Pharm.

[23] Bosman AW, Janssen HM, Meijer EW (1999). About dendrimers: structure, physical properties, and applications. Chem Rev.

[24] Jackson CL, Chanzy HD, Booy FP, Drake BJ, Tomalia DA, Bauer BJ, Amis EJ (1998). Visualization of dendrimer molecules by transmission electron microscopy (TEM): staining methods and cryo-TEM of vitrified solutions. Macromolecules.

[25] Menjoge AR, Kannan RM, Tomalia DA (2010). Dendrimer-based drug and imaging conjugates: design considerations for nanomedical applications. Drug Discov Today.

[26] Rao BN, Viswanath V, Reddy KR, Fathima SR, Surekha P, Bhuvaneswari S (2015). Dendrimers–structure, synthesis, encapsulation, characterization and application. J Global Trends Pharm Sci.

[27] Parekh HS (2007). The advance of dendrimers–a versatile targeting platform for gene/drug delivery. Curr Pharm Design.

[28] Kurtoglu YE, Mishra MK, Kannan S, Kannan RM (2010). Drug release characteristics of PAMAM dendrimer–drug conjugates with different linkers. Int J Pharm.

[29] Gupta V, Nayak S (2015). Dendrimers: A review on synthetic approaches. J Appl Pharm Sci.

[30] Liu J, Gray WD, Davis ME, Luo Y (2012). Peptide- and saccharide-conjugated dendrimers for targeted drug delivery: a concise review. Interface Focus.

[31] Yang H, Morris JJ, Lopina ST (2004). Polyethylene glycol–polyamidoamine dendritic micelle as solubility enhancer and the effect of the length of polyethylene glycol arms on the solubility of pyrene in water. J Colloid Interface Sci.

[32] Pooresmaeil M, Namazi H (2021). Advances in development of the dendrimers having natural saccharides in their structure for efficient and controlled drug delivery applications. Eur Polym J.

[33] Agrawal P, Gupta U, Jain NK (2007). Glycoconjugated peptide dendrimers-based nanoparticulate system for the delivery of chloroquine phosphate. Biomaterials.

[34] Sharma AK, Gupta L, Sahu H, Qayum A, Singh SK, Nakhate KT (2018). Chitosan engineered PAMAM dendrimers as nanoconstructs for the enhanced anti-cancer potential and improved in vivo brain pharmacokinetics of temozolomide. Pharm Res.

[35] Han M, Huang-Fu M-Y, Guo W-W, Guo N-N, Chen J, Liu H-N (2017). MMP-2-Sensitive HA end-conjugated poly(amidoamine) dendrimers via click reaction to enhance drug penetration into solid tumor. ACS Appl Mater Interfaces.

[36] Arima H, Motoyama K, Higashi T (2013). Sugar-appended polyamidoamine dendrimer conjugates with cyclodextrins as cell-specific non-viral vectors. Adv Drug Deliv Rev.

[37] Agarwal A, Gupta U, Asthana A, Jain NK (2009). Dextran conjugated dendritic nanoconstructs as potential vectors for anti-cancer agent. Biomaterials.

[38] He H, Yuan Q, Bie J, Wallace RL, Yannie PJ, Wang J (2018). Development of mannose functionalized dendrimeric nanoparticles for targeted delivery to macrophages: use of this platform to modulate atherosclerosis. Transl Res..

[39] Kolhe P, Khandare J, Pillai O, Kannan S, Lieh-Lai M, Kannan RM (2006). Preparation, cellular transport, and activity of polyamidoamine-based dendritic nanodevices with a high drug payload. Biomaterials.

[40] Lancina MG, Wang J, Williamson GS, Yang H (2018). DenTimol as a dendrimeric timolol analogue for glaucoma therapy: synthesis and preliminary efficacy and safety assessment. Mol Pharm.

[41] Lancina MG, Yang H (2017). Dendrimers for ocular drug delivery. Can J Chem.

[42] Wang G, Zhou Z, Zhao Z, Li Q, Wu Y, Yan S (2020). Enzyme-triggered transcytosis of dendrimer–drug conjugate for deep penetration into pancreatic tumors. ACS Nano.

[43] Jiang W, Luo X, Wei L, Yuan S, Cai J, Jiang X (2021). The sustainability of energy conversion inhibition for tumor ferroptosis therapy and chemotherapy. Small.

[44] Liu H, Wang H, Yang W, Cheng Y (2012). Disulfide cross-linked low generation dendrimers with high gene transfection efficacy, low cytotoxicity, and low cost. J Am Chem Soc.

[45] Shen Y, Zhou Z, Sui M, Tang J, Xu P, Kirk EAV (2010). Charge-reversal polyamidoamine dendrimer for cascade nuclear drug delivery. Nanomedicine.

[46] Pang X, Jiang Y, Xiao Q, Leung AW, Hua H, Xu C (2016). pH-Responsive polymer–drug conjugates: design and progress. J Control Release.

[47] Wang J, He H, Cooper RC, Gui Q, Yang H (2019). Drug-conjugated dendrimer hydrogel enables sustained drug release via a self-cleaving mechanism. Mol Pharm.

[48] Milhem OM, Myles C, McKeown NB, Attwood D, D’Emanuele A (2000). Polyamidoamine Starburst® dendrimers as solubility enhancers. Int J Pharm.

[49] Dufès C, Uchegbu IF, Schätzlein AG (2005). Dendrimers in gene delivery. Adv Drug Delivery Rev.

[50] Eliyahu H, Barenholz Y, Domb AJ (2005). Polymers for DNA delivery. Molecules.

[51] Surekha B, Kommana NS, Dubey SK, Kumar AVP, Shukla R, Kesharwani P (2021). PAMAM dendrimer as a talented multifunctional biomimetic nanocarrier for cancer diagnosis and therapy. Colloids Surface B.

[52] Choi YJ, Kang SJ, Kim YJ, Lim YB, Chung HW (2010). Comparative studies on the genotoxicity and cytotoxicity of polymeric gene carriers polyethylenimine (PEI) and polyamidoamine (PAMAM) dendrimer in Jurkat T-cells. Drug Chem Toxicol.

[53] Zhang J, Liu D, Zhang M, Sun Y, Zhang X, Guan G (2016). The cellular uptake mechanism, intracellular transportation, and exocytosis of polyamidoamine dendrimers in multidrug-resistant breast cancer cells. Int J Nanomed.

[54] Abedi-Gaballu F, Dehghan G, Ghaffari M, Yekta R, Abbaspour-Ravasjani S, Baradaran B (2018). PAMAM dendrimers as efficient drug and gene delivery nanosystems for cancer therapy. Appl Mater Today.

[55] Xu L, Yeudall WA, Yang H (2017). Folic acid-decorated polyamidoamine dendrimer exhibits high tumor uptake and sustained highly localized retention in solid tumors: Its utility for local siRNA delivery. Acta Biomater.

[56] Chaplot SP, Rupenthal ID (2013). Dendrimers for gene delivery–a potential approach for ocular therapy?. J Pharm Pharmacol.

[57] Cooper RC, Yang H (2020). Duplex of polyamidoamine dendrimer/custom-designed nuclear-localization sequence peptide for enhanced gene delivery. Bioelectricity.

[58] Al-Jamal KT, Al-Jamal WT, Kostarelos K, Turton JA, Florence AT (2012). Anti-angiogenic poly-L-lysine dendrimer binds heparin and neutralizes its activity. Results Pharma Sci.

[59] Kannan RM, Nance E, Kannan S, Tomalia DA (2014). Emerging concepts in dendrimer-based nanomedicine: from design principles to clinical applications. J Intern Med.

[60] Yang H (2016). Targeted nanosystems: advances in targeted dendrimers for cancer therapy. Nanomedicine.

[61] Madaan K, Kumar S, Poonia N, Lather V, Pandita D (2014). Dendrimers in drug delivery and targeting: drug-dendrimer interactions and toxicity issues. J Pharm Bioall Sci.

[62] Sonawane ND, Szoka FC, Verkman AS (2003). Chloride accumulation and swelling in endosomes enhances DNA transfer by polyamine-DNA polyplexes. J Biol Chem.

[63] Kesharwani P, Banerjee S, Gupta U, Amin MCIM, Padhye S, Sarkar FH (2015). PAMAM dendrimers as promising nanocarriers for RNAi therapeutics. Mater Today.

[64] Zhou L, Gan L, Li H, Yang X (2007). Studies on the interactions between DNA and PAMAM with fluorescent probe [Ru(phen)2d ppz]2+. J Pharmaceut Biomed.

[65] Thiagarajan G, Greish K, Ghandehari H (2013). Charge affects the oral toxicity of poly(amidoamine) dendrimers. Eur J Pharm Biopharm.

[66] Chauhan AS, Jain NK, Diwan PV (2010). Pre-clinical and behavioural toxicity profile of PAMAM dendrimers in mice. Proc R Soc A.

[67] de Araujo RV, Santos SS, Ferreira EI, Giarolla J (2018). New advances in general biomedical applications of PAMAM dendrimers. Molecules.

[68] Pryor JB, Harper BJ, Harper SL (2014). Comparative toxicological assessment of PAMAM and thiophosphoryl dendrimers using embryonic zebrafish. Int J Nanomedicine.

[69] Gao Y, Wang J, Chai M, Li X, Deng Y, Jin Q (2020). Size and charge adaptive clustered nanoparticles targeting the biofilm microenvironment for chronic lung infection management. ACS Nano.

[70] Wang K, Tu Y, Yao W, Zong Q, Xiao X, Yang R-M (2020). Size-switchable nanoparticles with self-destructive and tumor penetration characteristics for site-specific phototherapy of cancer. ACS Appl Mater Inter.

[71] Mohapatra A, Uthaman S, Park I-K, Kesharwani P, Paknikar KM, Gajbhiye V (2019). Polyethylene glycol nanoparticles as promising tools for anticancer therapeutics. Polymeric nanoparticles as a promising tool for anticancer therapeutics.

[72] Luong D, Kesharwani P, Deshmukh R, Amin MCIM, Gupta U, Greish K (2016). PEGylated PAMAM dendrimers: enhancing efficacy and mitigating toxicity for effective anticancer drug and gene delivery. Acta Biomater.

[73] Ho MN, Bach LG, Nguyen DH, Nguyen CH, Nguyen CK, Tran NQ, Nguyen NV, Thi TTH (2019). PEGylated PAMAM dendrimers loading oxaliplatin with prolonged release and high payload without burst effect. Biopolymers.

[74] Zhu S, Hong M, Zhang L, Tang G, Jiang Y, Pei Y (2009). PEGylated PAMAM dendrimer-doxorubicin conjugates: in vitro evaluation and in vivo tumor accumulation. Pharm Res.

[75] Yuan Q, Yeudall WA, Yang H (2010). PEGylated polyamidoamine dendrimers with bis-aryl hydrazone linkages for enhanced gene delivery. Biomacromol.

[76] Longley CB, Zhao H, Lozanguiez YL, Conover CD (2013). Biodistribution and excretion of radiolabeled 40 kDa polyethylene glycol following intravenous administration in mice. J Pharm Sci.

[77] Hu X, Olivier K, Polack E, Crossman M, Zokowski K, Gronke RS (2011). In vivo pharmacology and toxicology evaluation of polyethylene glycol-conjugated interferon β-1a. J Pharmacol Exp Ther.

[78] Gokay SS, Celik T, Sari YM, Ekinci F, Yildizdas RD, Yilmaz HL (2018). Urticaria as a rare side effect of polyethylene glycol-3350 in a child: case report. Acta Clin Croat.

[79] Abu Lila AS, Kiwada H, Ishida T (2013). The accelerated blood clearance (ABC) phenomenon: clinical challenge and approaches to manage. J Control Release.

[80] Im H-J, England CG, Feng L, Graves SA, Hernandez R, Nickles RJ (2016). Accelerated blood clearance phenomenon reduces the passive targeting of PEGylated nanoparticles in peripheral arterial disease. ACS Appl Mater Inter.

[81] Son K, Ueda M, Taguchi K, Maruyama T, Takeoka S, Ito Y (2020). Evasion of the accelerated blood clearance phenomenon by polysarcosine coating of liposomes. J Control Release.

[82] Waite CL, Sparks SM, Uhrich KE, Roth CM (2009). Acetylation of PAMAM dendrimers for cellular delivery of siRNA. BMC Biotech.

[83] Xiong Z, Shen M, Shi X (2019). Zwitterionic Modification of nanomaterials for improved diagnosis of cancer cells. Bioconjugate Chem.

[84] Wang L, Shi C, Wang X, Guo D, Duncan TM, Luo J (2019). Zwitterionic Janus dendrimer with distinct functional disparity for enhanced protein delivery. Biomaterials.

[85] Wang G, Wu B, Li Q, Chen S, Jin X, Liu Y (2020). Active transportation of liposome enhances tumor accumulation, penetration, and therapeutic efficacy. Small.

[86] Zhou Q, Shao S, Wang J, Xu C, Xiang J, Piao Y (2019). Enzyme-activatable polymer-drug conjugate augments tumour penetration and treatment efficacy. Nat Nanotechnol.

[87] Feng S, Zhang H, Zhi C, Gao XD, Nakanishi H (2018). pH-Responsive charge-reversal polymer-functionalized boron nitride nanospheres for intracellular doxorubicin delivery. Int J Nanomed.

[88] Hua Y, Chen L, Hou C, Liu S, Pei Z, Lu Y (2020). Supramolecular vesicles based on amphiphilic pillar[n]arenes for smart nano-drug delivery. Int J Nanomed.

[89] Zhou Z, Shen Y, Tang J, Jin E, Ma X, Sun Q (2011). Linear polyethyleneimine-based charge-reversal nanoparticles for nuclear-targeted drug delivery. J Mater Chem.

[90] Liu X, Xiang J, Zhu D, Jiang L, Zhou Z, Tang J (2016). Fusogenic reactive oxygen species triggered charge-reversal vector for effective gene delivery. Adv Mater.

[91] Wang G, Zhu D, Zhou Z, Piao Y, Tang J, Shen Y (2020). A glutathione-specific and intracellularly labile polymeric nanocarrier for efficient and safe cancer gene delivery. ACS Appl Mater Inter.

[92] Wang J, Cooper RC, Yang H (2020). Polyamidoamine dendrimer grafted with an acid-responsive charge-reversal layer for improved gene delivery. Biomacromol.

[93] Li J, Mooney DJ (2016). Designing hydrogels for controlled drug delivery. Nat Rev Mater.

[94] Wang J, Guo C, Wang X-Y, Yang H (2021). “Double-punch” strategy for delivery of viral immunotherapy with prolonged tumor retention and enhanced transfection efficacy. J Control Release.

[95] Ghobril C, Rodriguez EK, Nazarian A, Grinstaff MW (2016). Recent advances in dendritic macromonomers for hydrogel formation and their medical applications. Biomacromol.

[96] Desai PN, Yuan Q, Yang H (2010). Synthesis and characterization of photocurable polyamidoamine dendrimer hydrogels as a versatile platform for tissue engineering and drug delivery. Biomacromol.

[97] Wang J, He H, Cooper RC, Yang H (2017). In Situ-Forming Polyamidoamine dendrimer hydrogels with tunable properties prepared via aza-Michael addition reaction. ACS Appl Mater Inter.

[98] Cong H, Zhou L, Meng Q, Zhang Y, Yu B, Shen Y (2019). Preparation and evaluation of PAMAM dendrimer-based polymer gels physically cross-linked by hydrogen bonding. Biomater Sci.

[99] Cho IS, Ooya T (2018). A Supramolecular hydrogel based on polyglycerol dendrimer-specific amino group recognition. Chem Asian J.

[100] Soiberman U, Kambhampati SP, Wu T, Mishra MK, Oh Y, Sharma R (2017). Subconjunctival injectable dendrimer-dexamethasone gel for the treatment of corneal inflammation. Biomaterials.

[101] Xu L, Cooper RC, Wang J, Yeudall WA, Yang H (2017). Synthesis and application of injectable bioorthogonal dendrimer hydrogels for local drug delivery. ACS Biomater Sci Eng.

[102] Yang H, Tyagi P, Kadam RS, Holden CA, Kompella UB (2012). Hybrid dendrimer hydrogel/PLGA nanoparticle platform sustains drug delivery for one week and antiglaucoma effects for four days following one-time topical administration. ACS Nano.

[103] Wang J, Li B, Pu X, Wang X, Cooper RC, Gui Q (2020). Injectable multicomponent biomimetic gel composed of inter-crosslinked dendrimeric and mesoporous silica nanoparticles exhibits highly tunable elasticity and dual drug release capacity. ACS Appl Mater Inter.

[104] Wang J, Yang H (2018). Superelastic and pH-responsive degradable dendrimer cryogels prepared by cryo-aza-Michael addition reaction. Sci Rep.

[105] Wang Y, Zhao Q, Zhang H, Yang S, Jia X (2014). A novel poly(amido amine)-dendrimer-based hydrogel as a mimic for the extracellular matrix. Adv Mater.

[106] Cheng C-Y, Wang N, Wong TY, Congdon N, He M, Wang YX (2020). Prevalence and causes of vision loss in East Asia in 2015: magnitude, temporal trends and projections. Brit J Ophthalmol.

[107] El Hoffy NM, Abdel Azim EA, Hathout RM, Fouly MA, Elkheshen SA (2021). Glaucoma: management and future perspectives for nanotechnology-based treatment modalities. Eur J Pharm Sci.

[108] Cooper RC, Yang H (2019). Hydrogel-based ocular drug delivery systems: emerging fabrication strategies, applications, and bench-to-bedside manufacturing considerations. J Control Release.

[109] Wang J, Williamson GS, Lancina MG, Yang H (2017). Mildly cross-linked dendrimer hydrogel prepared via aza-Michael addition reaction for topical brimonidine delivery. J Biomed Nanotechnol.

[110] Wang J, Cooper RC, He H, Li B, Yang H (2018). Polyamidoamine dendrimer microgels: hierarchical arrangement of dendrimers into micrometer domains with expanded structural features for programmable drug delivery and release. Macromolecules.

[111] Wang J, Li B, Huang D, Norat P, Grannonico M, Cooper RC (2021). Nano-in-nano dendrimer gel particles for efficient topical delivery of antiglaucoma drugs into the eye. Chem Eng J.

